# Meta-analyses overgeneralize SGLT2 inhibitor effectiveness in population with low albuminuria, no diabetes, no heart failure and no atherovascular heart disease

**DOI:** 10.1093/ndt/gfag009

**Published:** 2026-01-22

**Authors:** Mariana Murea, Carlo Basile, Giorgina B Piccoli

**Affiliations:** Department of Internal Medicine, Section on Nephrology, Wake Forest University School of Medicine, Winston-Salem, NC, USA; Associazione Nefrologica Gabriella Sebastio, Martina Franca, Italy; Néphrologie et Dialyse, Centre Hospitalier Le Mans, Le Mans, France

To the Editor

Sodium–glucose cotransporter-2 (SGLT2) inhibitors have been analyzed in two recent SGLT2 Inhibitor Meta-Analysis Cardio-Renal Trialists’ Consortium (SMART-C) individual-participant meta-analyses [1, 2]. Both meta-analyses concluded that SGLT2 inhibitors improve patient outcomes across a broad range of baseline kidney function and albuminuria, including those in low urine albumin-to-creatinine ratio (UACR) strata, and urged removal of UACR stratification from clinical guidelines. We critically appraise the SMART-C meta-analyses for low-albuminuria chronic kidney disease (CKD), and delineate where certainty is insufficient to claim outcome benefit in all patients with CKD “irrespective” of albuminuria. Our analysis is strictly evidence-based (no re-estimation), anchored to published subgroup results and the eligibility landscape of contributing randomized clinical trials (RCTs), described in the Supplementary data, Table S1.

Neuen *et al*. (*n* = 70 361) and Staplin *et al*. (*n* = 58 816) pooled double-blind, placebo-controlled RCTs of SGLT2 inhibitors that spanned type 2 diabetes at high risk of atherosclerotic cardiovascular disease (ASCVD), CKD and heart failure (HF) [1, 2]. We extracted subgroup hazard ratios (HRs) and event rates as presented in the source publications and contrasted relative effects with absolute effects for patient outcomes in the relevant subgroups. We estimated absolute risk reduction (ARR) by applying the HR and its 95% confidence interval (CI) bounds to the placebo rate and expressed ARR per 100 patient-years; we then calculated the 1-year number needed to treat (NNT) as 100/ARR when the lower bound of ARR’s 95% CI was positive (otherwise, NNT was not estimable).

Participants were middle-aged (Neuen *et al*.: mean 64.8 years; Staplin *et al*.: mean 64 years) and had type 2 diabetes or HF, or were enrolled via albuminuric CKD criteria; CKD trials required moderate-to-severe albuminuria (e.g. UACR ≥200–300 mg/g) or reduced estimated glomerular filtration rate (eGFR) (Supplementary data, Table S1).

Relative effects (HRs) and absolute effects (ARR and 1-year NNT) are summarized in Tables 1 and 2, respectively. For the outcome of CKD progression, relative effect estimates at low-UACR categories in participants without diabetes were imprecise, with HR 1.15 (95% CI 0.59–2.22) for UACR <30 mg/g and 0.83 (95% CI 0.45–1.53) for UACR 30–300 mg/g (Table 1). The ARR for CKD progression in participants without diabetes and UACR <200 mg/g was 0.16 events per 100 patient-years (95% CI –0.30 to 0.47), corresponding to a non-estimable NNT over 1 year (Table 2). For any hospitalization, benefits were present in non-diabetics with UACR <200 mg/g (HR 0.89; 95% CI 0.83–0.95; ARR 3.13 per 100 patient-years; NNT ≈ 32) (Table 2). Hospitalization reductions were driven by HF-enriched populations as sensitivity analysis that excluded HF trials showed much smaller absolute effects (Supplementary data, Table S2). In Staplin *et al*., non-diabetic patients with UACR <200 mg/g had HR for any death of 0.93 (95% CI 0.79–1.10) with ARR 0.30 (95% CI −0.43 to 0.90) and non-estimable NNT; and HR for acute kidney injury of 0.74 (95% CI 0.54–1.01) with ARR 0.39 per 100 patient-years (95% CI −0.02 to 0.69) and non-estimable NNT (Table 2).

**Table 1:** Relative effects on CKD progression by baseline UACR in SMART‑C SGLT2 trials.

UACR category	SGLT2 events/participants	Placebo events/participants	HRs (95% CI)
All participants			
UACR <30 mg/g	94/15 973	125/13 595	0.58 (0.44–0.76)
UACR 30–300 mg/g	109/7583	128/6519	0.74 (0.57–0.96)
UACR >300 mg/g	637/7575	999/7275	0.57 (0.52–0.64)
With diabetes			
UACR <30 mg/g	73/13 984	108/11 645	0.52 (0.38–0.70)
UACR 30–300 mg/g	89/6365	103/5309	0.72 (0.53–0.96)
UACR >300 mg/g	503/5849	786/5497	0.57 (0.51–0.64)
Without diabetes			
UACR <30 mg/g	21/1988	17/1950	1.15 (0.59–2.22)
UACR 30–300 mg/g	20/1218	25/1210	0.83 (0.45–1.53)
UACR >300 mg/g	134/1726	213/1778	0.63 (0.51–0.79)

CKD progression was defined as kidney failure, ≥50% sustained decline in eGFR or death due to kidney failure. HRs and 95% CIs are inverse variance–weighted pooled estimates from two‑stage Cox models in the SMART‑C individual‑participant data meta‑analysis by Neuen *et al*. [1]. There was no evidence of effect modification by UACR (*P* for interaction = .49). Absolute event rates and NNT cannot be derived from these subgroup analyses because person-time denominators are not reported.

**Table 2:** Absolute effects by diabetes status and baseline UACR in SMART‑C SGLT2 trials.

Outcome	Diabetes status	Placebo rate	SGLT2 rate	ARR (point; 95% CI)	NNT (1-year)	NNT 95% CI
UACR <200 mg/g						
Kidney disease progression	No diabetes	1.30	1.14	0.16 (–0.30 to 0.47)	Not estimable	
	Diabetes	2.20	1.43	0.77 (0.55 to 0.97)	130	103–182
Any hospitalization	No diabetes	28.50	25.37	3.13 (0.57 to 5.13)	32	19–175
	Diabetes	19.90	17.91	1.99 (1.59 to 2.59)	50	39–63
Acute kidney injury	No diabetes	1.50	1.11	0.39 (–0.02 to 0.69)	Not estimable	
	Diabetes	1.10	0.80	0.30 (0.14 to 0.43)	333	233–714
Any death	No diabetes	4.30	4.00	0.30 (–0.43 to 0.90)	Not estimable	
	Diabetes	3.60	3.24	0.36 (0.07 to 0.61)	278	164–1429
UACR ≥200 mg/g						
Kidney disease progression	No diabetes	7.20	5.11	2.09 (1.15 to 2.88)	48	35–87
	Diabetes	7.70	5.00	2.70 (2.23 to 3.16)	37	32–45
Any hospitalization	No diabetes	34.80	30.28	4.52 (0.35 to 8.00)	22	12–286
	Diabetes	29.40	25.58	3.82 (2.35 to 5.29)	26	19–43
Acute kidney injury	No diabetes	1.50	1.05	0.45 (–0.10 to 0.81)	Not estimable	
	Diabetes	2.60	2.11	0.49 (0.13 to 0.81)	204	123–769
Any death	No diabetes	6.20	4.90	1.30 (–0.56 to 2.67)	Not estimable	
	Diabetes	6.80	5.30	1.50 (0.88 to 2.04)	67	49–114

Rates are expressed per 100 patient-years and reflect first events only. Absolute effects were derived by applying subgroup-specific HRs reported by Staplin *et al*. to the observed placebo event rates [2]. ARR equals placebo rate minus SGLT2 rate. The 95% CIs for ARR were calculated by applying the upper and lower bounds of the HR to the placebo rate. NNT were calculated as 100/ARR over a 1-year horizon and are shown only when the lower bound of the ARR CI is positive; otherwise, NNT is not estimable.

Overgeneralization beyond trial populations is widespread: a methodological review identified this issue in ≥50% of the 553 RCTs published in the top four highest ranking medical journals [3]. We submit that the SMART-C meta-analyses offer limited empirical support for extending routine SGLT2 inhibitor use to patients with low UACR and without comorbidities such as diabetes or HF, as these phenotypes were sparsely represented and effect estimates were imprecise.

Importantly, both meta-analyses used one- or two-variable at-a-time subgroup analyses which fail to account for joint or interacting factors [1, 2]. Interpretation of such analyses could yield misleading inferences if risk dimensions that guide clinical decisions are not evaluated through joint stratification and analyzed simultaneously [4]. Patients with concordant low-risk features (UACR <200–300 mg/g, eGFR ≥45 mL/min/1.73 m², no diabetes, no ASCVD, no HF) were effectively excluded from the contributing RCTs. This limits applicability to common clinical scenarios, including hematuria-predominant glomerular disease, inherited or genetic kidney disease in younger individuals with preserved kidney function, and structural or cystic kidney disease defined by imaging rather than albuminuria.

Furthermore, absolute reductions in event rates were modest in the low-UACR strata, despite the presence of at least one additional high-risk comorbidity: participants without diabetes either had HF, reduced eGFR or both; likewise, participants without HF either had diabetes, reduced eGFR or both. The editorial by Gregg *et al*. pointed out that the likelihood of achieving an absolute benefit of preventing one case of CKD progression from SGLT2 inhibitor treatment is ∼14-fold lower for patients with UACR <200 mg/g compared with those with UACR >200 mg/g, despite the presence of other notable comorbid conditions [5].

Our observations do not challenge the established efficacy of SGLT2 inhibitors in trial-represented populations and align with current guideline frameworks, which broadened eligibility while maintaining stratification for treatment individualization by eGFR, albuminuria and comorbidities—offering Class 1A recommendations for most CKD groups and a weaker Class 2B recommendation specifically for patients with eGFR 20–45 mL/min/1.73 m² and UACR <200 mg/g without diabetes [6–8]. Nevertheless, the SMART-C authors assert that their findings “offer an opportunity for the guidelines to be simplified to reduce arguably unnecessary stratification of recommendations by diabetes status and by UACR […].” Indeed, one could argue that, from a population and lifetime risk standpoint, earlier initiation of SGLT2 inhibitors could plausibly accrue dialysis-free years if benefits translate into fewer hard kidney outcomes over time. However, for patients with UACR <200 mg/g and eGFR ≥45 mL/min/1.73 m² without diabetes or HF, long-term hard outcome and cost-effectiveness data are currently lacking. While absence of data does not equate to evidence against benefit, in these lower risk scenarios, treatment should be individualized, balancing potential benefits and harms, patient preferences, and contemporary considerations of drug affordability and access.

In summary, these SMART-C meta-analyses demonstrated consistent clinical benefit with SGLT2 inhibitor use across UACR and eGFR strata in patients with type 2 diabetes or HF, and in those with moderate to severe albuminuria (UACR ≥200–300 mg/g) without diabetes or HF. In contrast, patients with low UACR and eGFR ≥45 mL/min/1.73 m² without diabetes or HF were minimally represented, and short-term absolute effects remain uncertain. Additional studies are needed to determine whether SGLT2 inhibitors deliver hard outcome benefits and favorable value in low-risk and low-morbidity CKD phenotype.

## Supplementary Material

gfag009_Supplemental_File
