# Supplementary material for: Meta-analyses overgeneralize SGLT2 inhibitor effectiveness in population with low albuminuria, no diabetes, no heart failure and no atherovascular heart disease
Source: Nephrol Dial Transplant. 2026 Jan 22;41(6):1171–3. doi: 10.1093/ndt/gfag009 (PMC13270309; doi:10.1093/ndt/gfag009)
Supplement: gfag009_Supplemental_File [file gfag009_Supplemental_File.docx]

**Supplementary Material**

[Main Article’s Title]

**Metaanalyses Overgeneralize SGLT2 Inhibitor Effectiveness in Population with Low Albuminuria, No Diabetes, No Heart Failure, and No Atherovascular Heart Disease**

[Authors]

**Mariana Murea, MD,^1^** **Carlo Basile, MD^2^,** and **Giorgina B. Piccoli, MD^3^**

[Affiliations]

^1^Department of Internal Medicine, Section on Nephrology, Wake Forest University School of Medicine, Winston-Salem, North Carolina, USA.

^2^Associazione Nefrologica Gabriella Sebastio, Martina Franca, Italy.

**^3^**Néphrologie et Dialyse, Centre Hospitalier Le Mans, Le Mans, France.

**Contents**

[**Supplementary Methods** 2](#_Toc218073151)

[**Supplementary References** 3](#_Toc218073152)

[**Table S1. Eligibility Criteria of Randomized SGLT2 Inhibitor Trials Included in SMART‑C** 4](#_Toc218073153)

[**Table S2. Absolute Effects for Any Hospitalization in Non‑HF Trials (SMART‑C)** 5](#_Toc218073154)

# **Supplementary Methods**

We limited our analysis to SMART‑C sources and did not perform economic modelling or guideline grading; conclusions are bounded by reported hard outcomes and subgroup precision. We used published aggregate data with no access to individual participation data; institutional ethics approval was not required. Neuen et al. study was a patient‑level (individual participant data) meta‑analysis [1]; Staplin et al. employed a hybrid approach: patient‑level data for the empagliflozin trials and aggregate (summary) data for the other randomized clinical trials (RCTs) [2].

Neuen et al. analyzed 10 randomized, double-blind, placebo-controlled trials (n=70,361) evaluating SGLT2 inhibitors with label indications for reducing chronic kidney disease (CKD) progression. The trials included three in patients with type 2 diabetes and atherosclerotic cardiovascular disease (ASCVD), four in patients with heart failure (HF) across ejection fraction spectrum, and three CKD outcome trials. Treatment effects were stratified by baseline eGFR (≥60, 45–<60, 30–<45, <30 mL/min/1.73 m²) and by UACR (<30, 30–300, >300 mg/g). The primary outcome was CKD progression, defined as kidney failure (dialysis, transplant, or eGFR <15 mL/min/1.73 m²), ≥50% reduction in eGFR, or death due to kidney failure [1].

Staplin et al. is a subset of Neuen’s: it includes 8 of the same trials but omits DAPA‑HF and DELIVER because those trials did not collect baseline UACR. It pooled three RCTs of type 2 diabetes trials with high risk of ASCVD, two RCTs of HF, and three RCTs of CKD (n=58,816; 48,946 with diabetes, 9,870 without). Patients were stratified first by diabetes status (with vs without diabetes) and then by UACR threshold (≥200 vs <200 mg/g), requiring baseline UACR for inclusion. Key efficacy outcomes (evaluated without hierarchy) included: (i) kidney disease progression (composite of sustained ≥40% eGFR decline, kidney failure [dialysis, transplantation, or sustained eGFR <15 mL/min/1.73 m²], or kidney death); (ii) acute kidney injury (MedDRA Preferred Term); (iii) hospitalization for heart failure; (iv) all-cause hospitalization; and (v) all-cause death [2].

Disease-specific eligibility criteria, kidney function thresholds, albuminuria requirements, and renin–angiotensin system blockade mandates for each trial are summarized in **Table S1**. Where Staplin et al. reported subgroup placebo rates (per 1000 patient‑years) and diabetes/UACR‑specific HRs, we estimated absolute risk reduction (ARR) and then calculated the 1‑year number needed to treat (NNT) as 100/ARR when the lower bound of ARR’s 95% CI was positive (otherwise, NNT was not estimable) [2]. For Neuen et al., subgroup person‑time denominators for albuminuria strata were not available; therefore, ARR/NNT were not derived from that analysis [1]. Positive ARR indicates fewer events with SGLT2 inhibitors vs placebo; negative ARR indicates more events. Statistical significance depends on whether the ARR 95% CI excludes 0. The ARR point value is a magnitude marker. For example, an ARR of 1.0 per 100 patient‑years indicates one event is avoided per 100 patient‑years. Where the ARR 95% CI includes 0, the NNT is not estimable (the denominator would approach infinity). When ARR’s 95% CI is fully positive, we report NNT 95% CI as [100/ARR_upper, 100/ARR_lower].

# **Supplementary References**

**S1.** Zinman B, Wanner C, Lachin JM, et al. Empagliflozin, cardiovascular outcomes, and mortality in type 2 diabetes. N Engl J Med. 2015;373(22):2117–2128. doi:10.1056/NEJMoa1504720.

**S2.** Neal B, Perkovic V, Mahaffey KW, et al. Canagliflozin and cardiovascular and renal events in type 2 diabetes. N Engl J Med. 2017;377(7):644–657. doi:10.1056/NEJMoa1611925.

**S3.** Wiviott SD, Raz I, Bonaca MP, et al. Dapagliflozin and cardiovascular outcomes in type 2 diabetes. N Engl J Med. 2019;380(4):347–357. doi:10.1056/NEJMoa1812389.

**S4.** McMurray JJV, Solomon SD, Inzucchi SE, et al. Dapagliflozin in patients with heart failure and reduced ejection fraction. N Engl J Med. 2019;381(21):1995–2008. doi:10.1056/NEJMoa1911303.

**S5.** Packer M, Anker SD, Butler J, et al. Cardiovascular and renal outcomes with empagliflozin in heart failure. N Engl J Med. 2020;383(15):1413–1424. doi:10.1056/NEJMoa2022190.

**S6.** Anker SD, Butler J, Filippatos G, et al. Empagliflozin in heart failure with a preserved ejection fraction. N Engl J Med. 2021;385(16):1451–1461. doi:10.1056/NEJMoa2107038.

**S7.** Solomon SD, McMurray JJV, Claggett B, et al. Dapagliflozin in heart failure with mildly reduced or preserved ejection fraction. N Engl J Med. 2022;387(12):1089–1098. doi:10.1056/NEJMoa2206286.

**S8.** Perkovic V, Jardine MJ, Neal B, et al. Canagliflozin and renal outcomes in type 2 diabetes and nephropathy. N Engl J Med. 2019;380(24):2295–2306. doi:10.1056/NEJMoa1811744.

**S9.** Heerspink HJL, Stefánsson BV, Correa‑Rotter R, et al. Dapagliflozin in patients with chronic kidney disease. N Engl J Med. 2020;383(15):1436–1446. doi:10.1056/NEJMoa2024816.

**S10.** EMPA‑KIDNEY Collaborative Group; Herrington WG, Staplin N, Wanner C, et al. Empagliflozin in patients with chronic kidney disease. N Engl J Med. 2023;388(2):117–127. doi:10.1056/NEJMoa2204233.

# **Table S1. Eligibility Criteria of Randomized SGLT2 Inhibitor Trials Included in SMART‑C**

| **Table S1**. **Eligibility Criteria of Randomized SGLT2 Inhibitor Trials Included in SMART‑C** | | | | | | | |
| --- | --- | --- | --- | --- | --- | --- | --- |
| **Trial (drug) [ref]** | **Disease domain** | **eGFR inclusion criterion** | **UACR inclusion criterion** | **Disease-specific inclusion criteria** | **RAS blockade required** | **Notes relevant to low-UACR representation** | |
| EMPA-REG OUTCOME (empagliflozin) [S1] | Type 2 diabetes / ASCVD | ≥ 30 | Not required | Type 2 diabetes with established ASCVD | No | Albuminuria not an entry criterion; CV-risk trial |  |
| CANVAS Program (canagliflozin)  [S2] | Type 2 diabetes / high CV risk | ≥ 30 | Not required | Type 2 diabetes with ASCVD or ≥2 CV risk factors | No | Albuminuria not required; CV-risk enriched |  |
| DECLARE–TIMI 58 (dapagliflozin)  [S3] | Type 2 diabetes / high CV risk | ≥ 60 (CrCl) | Not required | Type 2 diabetes with ASCVD or CV risk factors | No | Higher baseline kidney function |  |
| DAPA-HF (dapagliflozin)  [S4] | Heart failure (HFrEF) | ≥ 30 | Not collected | NYHA II–IV; LVEF ≤40% | No | Excluded from UACR analyses |  |
| DELIVER (dapagliflozin)  [S5] | Heart failure (HFpEF) | Not specified | Not collected | HF with LVEF >40% | No | Excluded from UACR analyses |  |
| EMPEROR-Reduced (empagliflozin)  [S6] | Heart failure (HFrEF) | ≥ 20 | Not required | NYHA II–IV; LVEF ≤40% | No | HF-enriched population |  |
| EMPEROR-Preserved (empagliflozin)  [S7] | Heart failure (HFpEF) | ≥ 20 | Not required | HF with LVEF >40% | No | HF-enriched population |  |
| CREDENCE (canagliflozin)  [S8] | CKD (diabetic nephropathy) | 30–90 | 300–5000 | Albuminuric CKD with diabetes | Yes | Excludes low-UACR CKD |  |
| DAPA-CKD (dapagliflozin)  [S9] | CKD (with/without diabetes) | 25–75 | 200–5000 | Albuminuric CKD | Yes | Requires UACR ≥200 |  |
| EMPA-KIDNEY (empagliflozin)  [S10] | CKD (with/without diabetes) | 20–<45 any UACR  *or*  45–<90 with UACR ≥200 | Conditional | Broad CKD etiologies | Yes* | Low-UACR allowed only if eGFR <45 |  |
| Note: Neuen et al. included all the 10 RCTs [1]. Staplin et al. excluded DAPA-HF and DELIVER due to absence of baseline UACR collection [2]. ASCVD denotes atherosclerotic cardiovascular disease; CKD, chronic kidney disease; eGFR, estimated glomerular filtration rate (mL/min/1.73 m²); HF, heart failure; HFrEF, heart failure with reduced ejection fraction; HFpEF, heart failure with preserved ejection fraction; RAS, renin–angiotensin system; UACR = urinary albumin-to-creatinine ratio (mg/g). | | | | | | | |

# **Table S2. Absolute Effects for Any Hospitalization in Non‑HF Trials (SMART‑C)**

| **Table S2**. **Absolute Effects for Any Hospitalization in Non‑HF Trials (SMART‑C)** | | | | | | |
| --- | --- | --- | --- | --- | --- | --- |
| Diabetes status | Placebo rate | SGLT2i rate | ARR per 100 pt-yrs | ARR 95% CI (per 100 pt-yrs) | NNT (1-yr) | NNT 95% CI |
| **UACR <200 mg/g** | | | | | | |
| Diabetes | 11.12 | 10.12 | 1.00 | 0.67 to 1.33 | 100 | 75 to 150 |
| No diabetes | 14.62 | 13.01 | 1.61 | 0.73 to 2.48 | 62 | 40 to 137 |
| **UACR ≥200 mg/g** | | | | | | |
| Diabetes | 15.35 | 13.36 | 2.00 | 1.23 to 2.76 | 50 | 36 to 81 |
| No diabetes | 10.54 | 9.17 | 1.37 | 0.11 to 2.42 | 73 | 41 to 949 |
| Note: Rates are expressed per 100 patient-years and reflect first events only. Absolute effects were derived by applying subgroup-specific hazard ratios (HRs) for any hospitalization from Staplin et al. to the average placebo event rate computed from non–heart-failure trials only, reconstructing placebo events as E_placebo ≈ E_total/(1+HR) and placebo patient-years as (participants/2) × median follow-up per trial [2]. Values are shown per 100 patient-years; NNT is 1-year and reported only when the lower bound of ARR’s 95% CI > 0. | | | | | | |
